# Identifying exercise and cognitive intervention parameters to optimize executive function in older adults with mild cognitive impairment and dementia: a systematic review and meta-analyses of randomized controlled trials

**DOI:** 10.1186/s11556-024-00357-4

**Published:** 2024-08-30

**Authors:** Wenxin Chen, Jessie Leuk Siew-Pin, Yuhang Wu, Ning Huang, Wei-Peng Teo

**Affiliations:** 1 Physical Education College, Hubei University of Arts and Sciences, Hubei, China; 2 Physical Education and Sport Science (PESS) Department, National Institute of Education, Nanyang Technological University, Singapore, Singapore; 3 School of Transportation and Logistics Engineering, Wuhan University of Technology, Wuhan, China; 4 School of Public Health, Peking University, Beijing, China; 5 Science of Learning in Education Centre (SoLEC), National Institute of Education, Nanyang Technological University, 1 Nanyang Walk, Singapore, 637616 Singapore

**Keywords:** Aerobic exercise, Dual-task training, Executive functioning, Mind-body exercise, Resistance training

## Abstract

Physical exercise is recognized for its beneficial effects on brain health and executive function, particularly through the careful manipulation of key exercise parameters, including type, intensity, and duration. The aim of this systematic review and meta-analysis was to delineate the optimal types, intensities, and durations of exercise that improve cognitive functions in older adults with mild cognitive impairment (MCI) or dementia. A comprehensive search was conducted in Scopus, Web of Science, and PubMed from their inception until December 2023. The methodological quality and publication bias of the included studies were assessed using the PEDro scale and Egger’s regression test, respectively. Separate meta-analyses were performed to assess the overall impact of exercise on cognitive assessments and to explore the effects of different exercise types (i.e., aerobic, resistance, dual-task, mind-body, and multi-component exercises) and intensities (i.e., low, moderate, and high) on executive function. Results were presented as standardized mean differences (SMD) and 95% confidence intervals (95% CI). A meta-regression analysis was conducted to examine the correlation between exercise duration and mean effects. In total, 15,087 articles were retrieved from three databases, of which 35 studies were included in our final analyses. The results indicated high overall methodological quality (PEDro score = 8) but a potential for publication bias (t = 2.08, *p* = 0.045). Meta-analyses revealed that all types of exercise (SMD = 0.691, CI [0.498 to 0.885], *p* < 0.001) and intensities (SMD = 0.694, CI [0.485 to 0.903], *p* < 0.001) show significant effects favoring exercise. Notably, dual-task exercises (SMD = 1.136, CI [0.236 to 2.035], *p* < 0.001) and moderate-intensity exercises (SMD = 0.876, CI [0.533 to 1.219], *p* < 0.001) exhibited the greatest effect. No significant correlation was observed between exercise duration and SMD (R² = 0.038, *p* = 0.313). Overall, our meta-analyses support the role of physical exercise in enhancing executive function in older adults with MCI or dementia. It is essential to carefully tailor exercise parameters, particularly type and intensity, to meet the specific needs of older adults with MCI or dementia. Such customization is crucial for optimizing executive function outcomes and improving overall brain health.

**Supplementary Information:**

The online version contains supplementary material available at 10.1186/s11556-024-00357-4.

## Introduction

With advancements in global medicine and healthcare, there has been a significant increase in average human life expectancy. This, combined with declining birthrates, has contributed to a growing aging population across developed and developing nations worldwide [1]. The World Health Organization (WHO) estimates that the global population over the age of 60 years would reach 2.1 billion by 2050 [2, 3]. Consequently, this demographic shift is associated with an increase in chronic neurodegenerative conditions such as mild cognitive impairment (MCI) and dementia, primarily affecting older adults [4–7]. Annually, the number of individuals affected by MCI and dementia increases, with an estimated 139 million people projected to live with dementia worldwide by 2025, as reported in the World Alzheimer Report (2023) [8]. Although physical and cognitive functions decline with age, it is important to recognize that MCI and dementia are not inevitable consequences of aging [9]. These conditions are characterized by abnormal changes in brain structure and function [10], resulting in significant declines in memory, language and communication skills, motor function, mood, and social behavior [11, 12]. To date, no pharmacological interventions have proven effective at reversing the progression of MCI or dementia [13–15]. Therefore, a combination of pharmacological and non-pharmacological approaches, including sleep, diet, physical, and cognitive exercises, have become crucial early strategies to mitigate cognitive decline in older adults.

Physical exercise is known to positively affect various bodily systems, including the musculoskeletal, cardiovascular, metabolic, and central nervous systems [16, 17]. Moreover, physical exercise influences the brain through mechanisms, such as increasing cerebral blood flow, upregulating neurotrophic factors (i.e., brain-derived neurotrophic factors and insulin-like growth factor-1) [18, 19], promoting the release of neurotransmitters (i.e., dopamine and serotonin), and enhancing muscle-brain interactions [20, 21]. Additionally, most exercises are performed socially, fostering interaction, collaboration, and pro-social behaviors among participants [22]. These social activities are likely to improve cognitive function and reduce social isolation in older adults [23].

While physical exercise is known to benefit brain health, identifying the optimal exercise parameters to maximize these benefits is a critical question. Like other bodily training, achieving neural adaptations from exercise necessitates careful consideration of parameters (type, intensity, and duration) when designing interventions [12, 24, 25]. Several types of exercise, such as aerobic [26], resistance [27], dual-task [28], multi-component [29], and mind-body exercises [30], are proven to enhance cognitive function. However, the mechanisms by which executive function is enhanced may vary among different types of exercise. For example, aerobic exercise enhances cardiorespiratory and cardiovascular function, potentially lowering the risk of vascular dementia and cognitive decline associated with cerebral small vessel disease [31, 32]. Resistance and dual-task training, emphasizing functional movement and balance, demand effective cognitive processing [28, 33–35]. Evidence also suggests an inverted U-shaped relationship between exercise intensity and cognitive function [20, 29, 36], where moderate intensity enhances arousal and cognitive performance, whereas high intensity might impair it due to overstimulation or fatigue (i.e., hyperarousal states) and/or fatigue [34, 36, 37]. However, this inverted U-shaped relationship between exercise intensity and executive function has largely been observed only in healthy populations under acute exercise situations.

Given the complexity and variety of criteria used to assess executive function in individuals with MCI or dementia, the precise mechanisms by which exercise parameters (i.e., exercise type, intensity, and duration) impact executive function in older adults with MCI and dementia are still not fully understood. Identifying and tailoring optimal exercise parameters is crucial to ensure older adults with MCI or dementia derive optimal benefits through physical exercise, which supports not only physical health but also brain and cognitive health. Therefore, this systematic review and meta-analysis aim to synthesize the existing literature on the effects of exercise on executive function in older adults with MCI or dementia, taking into account exercise moderators such as exercise type, intensity, and duration. The research questions guiding this systematic review and meta-analysis are as follows:

1. Which type of physical exercise (i.e., aerobic, resistance, dual-tasking, and mind-body training) is most beneficial for executive function in older adults with MCI or dementia?
2. Which physical exercise intensity level (i.e., low, moderate, and high) is most beneficial for improving executive function in older adults with MCI or dementia?
3. What is the relationship between exercise duration and changes in executive function in older adults with MCI or dementia?

## Methods

### Study design and registration

**Fig. 1:**
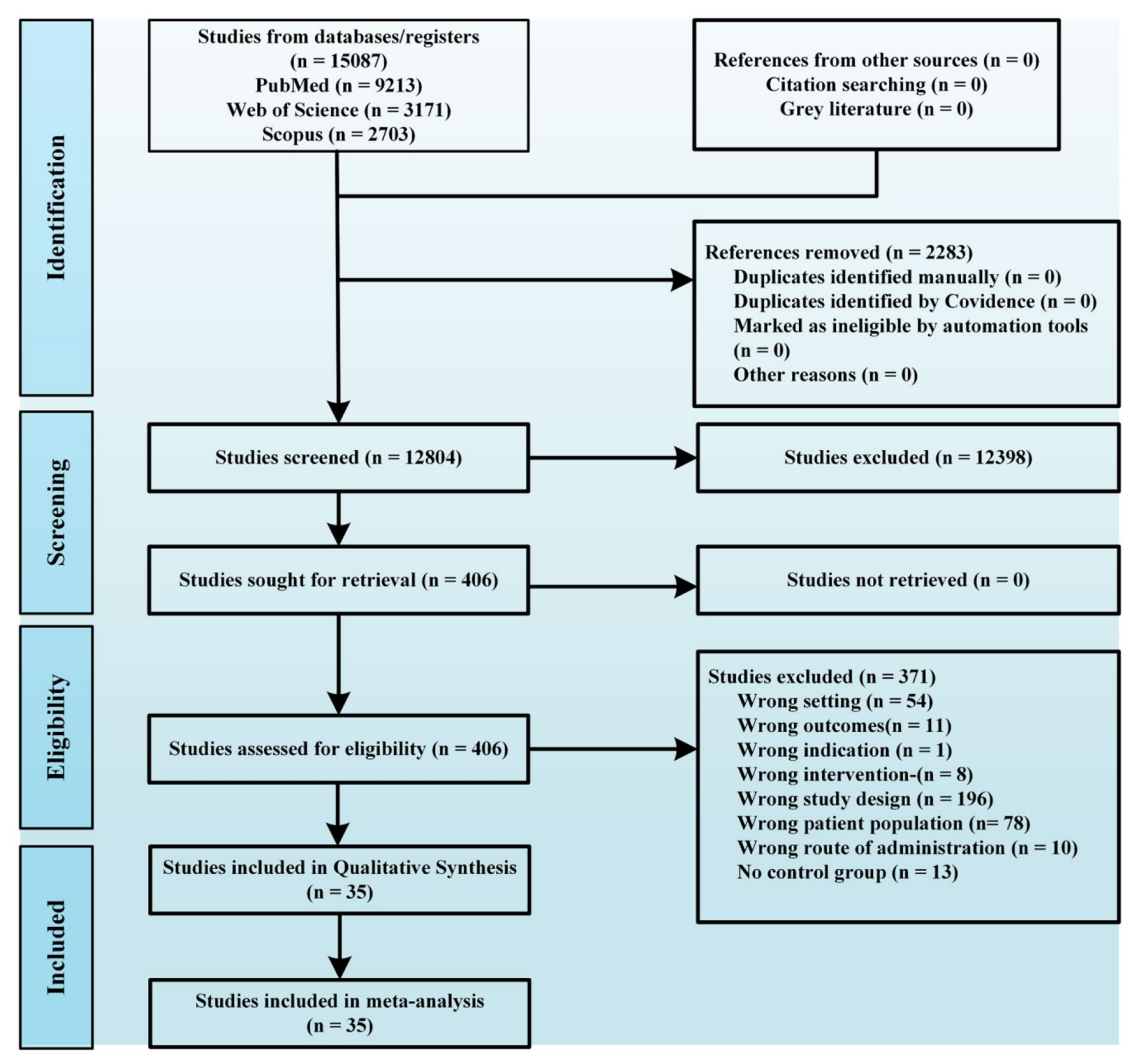
PRISMA flow diagram of study inclusion for this systematic review and meta-analysis

The systematic review and meta-analyses followed the Preferred Reporting Items for Systematic Reviews and Meta-Analysis (PRISMA) guidelines (Fig. 1) and is registered with the International Prospective Register of Systematic Reviews (XXX).

### Literature search strategy

A systematic literature search was conducted using three databases: PubMed, Web of Science, and Scopus, from inception to December 2023. The search terms were collaboratively developed by the authors, drawing on recent systematic reviews related to similar topics. Keywords were derived using the PICO framework, which includes participant/patient, intervention, comparator/comparison, and outcomes. The search strings comprised terms such as: (‘Alzheimer’s disease’ OR ‘mild cognitive impairment’ OR ‘dementia’ OR ‘AD’ OR ‘MCI’) AND (‘Aerobic exercise’ OR ‘resistance exercise’ OR ‘dual-task training’ OR ‘cognitive-motor training’ OR ‘motor-motor training’ OR ‘strength training’ OR ‘physical training’ OR ‘cardiovascular exercise’ OR ‘Yoga’ OR ‘mind-body exercise’ OR ‘multicomponent exercise’ OR ‘Taichi’ OR ‘Baduanjin’) AND (‘executive function’ OR ‘cognitive function’ OR ‘cognitive abilities’ OR ‘working memory’ OR ‘inhibition’ OR ‘attention’). Furthermore, references from existing systematic reviews and meta-analyses, along with the studies included in these reviews, were examined to identify further relevant studies.

### Study inclusion and exclusion criteria

Initially, 15,087 articles were retrieved from three databases and managed using the reference management software Covidence (Melbourne, Australia). After excluding 2,283 duplicates, 12,398 articles underwent screening based on titles and abstracts, resulting in 406 full-text articles. Following a detailed assessment, 371 articles were excluded, leaving 35 for inclusion in the systematic review and meta-analysis. Each article was independently evaluated by two researchers at each stage, with any disagreements resolved by discussion and consensus. The final list of included studies was approved by all authors, adhering to the following inclusion criteria:

1. The study included older adults with Alzheimer’s disease and MCI;
2. Interventions consisted of any organized exercise form (aerobic, resistance, dual-tasking, mind-body training), either acutely (single-session to < 8 weeks) or chronically (> 8 weeks);
3. Studies employed a randomized control design, with the control group performing routine programs such as usual-care treatments, simple motion exercises, or stretching at low-intensity;
4. Primary outcome measures were standardized cognitive neuropsychology assessment tests, such as but not limited to, the Mini-Mental State Examination (MMSE), Montreal Cognitive Assessment (MoCA) [38], Alzheimer’s Disease Assessment Scale–Cognitive Subscale (ADAS-Cog) [39], or dual-tasking performance [40, 41].
5. Articles had to be written in English and published as full-text in peer-reviewed journals.

Figure 1 depicts the study inclusion process, highlighting how studies were excluded based on irrelevant titles or abstracts failing to meet the inclusion criteria. When titles or abstracts were unclear, the complete article was subjected to review. The final selection of literature for discussion in the full text received unanimous approval from all authors. For additional details on the included studies, refer to Supplementary (S1).

### Identifying exercise parameters

The primary objective of this study is to assess the impact of exercise parameters on executive function in older adults with MCI or dementia. For subgroup analyses in the meta-analysis, the exercise parameters examined included exercise type, intensity, and duration. The definitions for each exercise parameter were derived from the guidelines provided by the American College of Sports Medicine (ACSM) [42, 43], essential for classifying and specifying the type, intensity, and duration of exercise in each study. For instance, exercise types were categorized according to their primary movements and intended goals, as outlined below:

1. ***Aerobic exercises***: These involve continuous and sustained activity over a period of time that targets cardiovascular function including, but not limited to walking, jogging, cycling, or swimming.
2. ***Resistance exercises***: These focus on muscle strengthening and require pushing or pulling against a resistance provided by either a machine, free weight, resistance band, or bodyweight.
3. ***Mind-body exercises***: These emphasize the connection between meditation or mindfulness and physical movement, including Tai Chi, Qi Gong, Baduanjin, and Yoga.
4. ***Dual-tasking exercises***: These involve performing two different tasks simultaneously, such as combining a motor task with a cognitive task or performing two motor tasks together.
5. ***Multicomponent exercises***: These consist of performing two different types of exercises sequentially, either within a single session or across sessions. This could include combining aerobic exercises with resistance exercises or other forms of exercise.

Exercise intensity evaluation utilized a combination of objective, subjective, and descriptive measures [44], detailed as follows:

1. ***Low***: Exercise that did not noticeably increase breathing rate and had a low energy requirement (< 3 METs, < 55% HR_max_, < 40% HRR, < 40% VO_2max_, PRE(C) < 10, PRE(C-R) ≤ 2) (METs: metabolic equivalents; HR_max_: heart rate maximum; HRR: heart rate recovery; VO_2max_: maximum oxygen consumption; Borg’s RPE scales C = category scale [6–20] and C-R = category-ratio scale [0–10]).
2. ***Moderate***: Exercises that could be performed while maintaining an uninterrupted conversation and typically lasted between 30 and 60 min (3–6 METs, 55–70% HR_max_, 40–60% HRR, 40–60% VO_2max_, PRE(C): 11–13, PRE(C-R): 3–4).
3. ***High***: Exercises that made it difficult to maintain an uninterrupted conversation and usually lasted less than 30 min (≥ 6 METs, ≥ 70% HR_max_, ≥ 60% HRR, ≥ 60% VO_2max_, PRE(C) ≥ 14, PRE(C-R) ≥ 5).

Lastly, exercise duration was the product of the number of exercise sessions per week, the duration of each exercise session, and the total number of weeks. For example, an exercise program that includes 3 sessions per week for 18 weeks, with each session lasting 30 min, would have a total duration of 1620 min (30 min/session × 2 sessions/week × 8 weeks).

### Methodological quality and bias assessment

Two reviewers evaluated the methodological quality of all studies using the Physiotherapy Evidence Database (PEDro) rating scale, which scores from 1 to 11. This scale assesses studies across five domains: group allocation, blinding, attrition, statistical analysis, and data variability. Ratings were assigned as “Yes” for supervised studies and “No” for items that were not applicable. Any discrepancies in ratings were resolved by a third reviewer. Methodological quality was categorized as Low (< 5), Good (6–8), and Excellent (9–10) [45].

### Data extraction

All retrieved titles and abstracts were imported into the reference management software, Covidence (Melbourne, Australia). After duplicate removal, two researchers independently screened the study titles and abstracts to identify studies potentially relevant for full-text retrieval. The full texts of relevant studies were independently reviewed by two researchers, who also examined potentially relevant articles in the reference lists. Two researchers extracted study characteristics, including first author, country, year of publication, population, design, number of participants, details of intervention and control groups (type, intensity, duration), and outcome measures for motor and cognitive functioning. Discrepancies in study selection or data extraction were resolved through discussions with a third researcher, and authors were contacted for additional information as necessary. Extracted data included domain-specific cognitions, categorized by researchers, encompassing pre- and post-intervention stimuli and quantitative data for the control (sham) condition, derived from text, tables, and graphs in each included study.

### Statistical analyses

Random effects meta-analyses were conducted to account for systematic influences and random errors between study-level effect sizes; results were displayed in forest plots showing averaged standardized mean differences (SMDs) and 95% confidence intervals (95%CI) [45, 46]. Positive SMD values signified that the intervention group outperformed the control group in cognitive tests for the outcome variables [47–49]. Separate meta-analyses on executive function outcome measures were carried out to investigate the impact of exercise on different outcomes. Subgroup meta-analyses were conducted to explore the relationship between exercise type and intensity and SMDs, as agreed upon a-priori to assess the influence of exercise parameters on executive functioning [50]. The exercise parameters considered for subgroup analyses included:

1. ***Exercise type*** – Aerobic vs. resistance vs. mind-body vs. dual-task vs. multicomponent exercises;
2. ***Exercise intensity*** – Low vs. moderate vs. high.

The *I*^*2*^ statistic was employed to assess statistical heterogeneity, with cut-off points corresponding to low (25%), moderate (50%), and high (75%) heterogeneity [51]. Funnel plots were used to evaluate publication bias via Egger’s regression test, where non-significant asymmetry suggested no bias [52]. Additionally, a meta-regression was performed to explore the effects of exercise duration on cognitive function, to determine whether exercise duration could predict the SMD of each study. All statistical analyses were performed using Comprehensive Meta-Analysis (V3.0, Biostat, Englewood, USA), with an alpha level of *P* < 0.05 to determine significance

## Results

### Overall studies

Thirty-five studies, spanning from 2010 to 2023 [6, 7, 12, 14, 15, 20, 24–37, 40, 41, 53–65], were included in the analysis, with 65% (*n* = 23) published after 2018 [7, 12, 14, 15, 20, 24, 28, 29, 31, 35–37, 40, 53–59, 63–65]. These studies targeted older adults with MCI, MD, or cognitive decline, involving sample sizes ranging from 27 to 280 participants, aged between 60 and 92 years, with a mean age of 75.09 ± 6.13 years (see Supplementary Table S1).

**Fig. 2:**
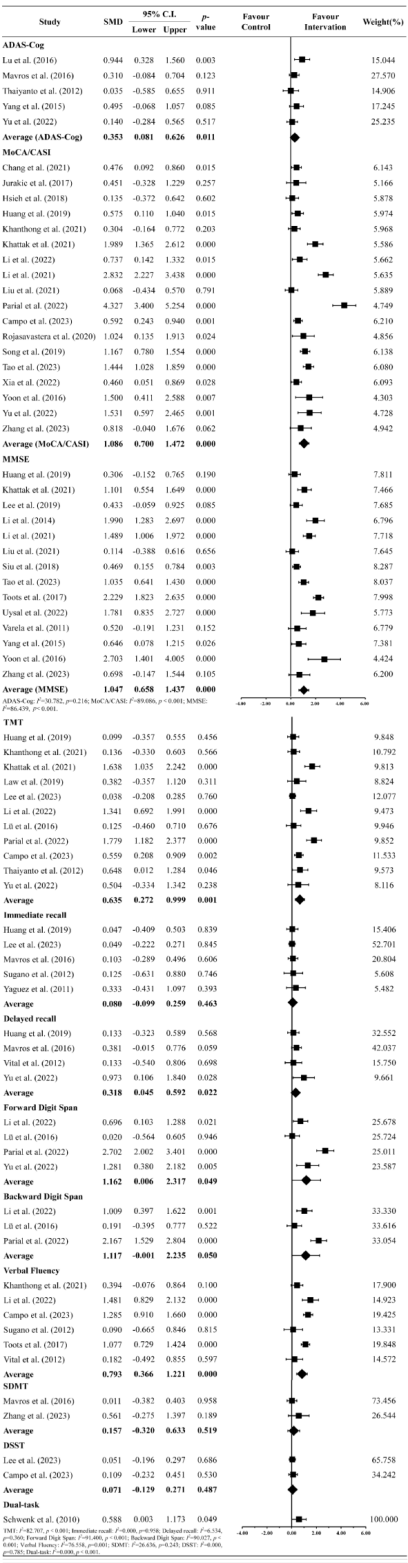
Forest plot of the overall effects of exercise on the various standardized clinical tests and assessments of cognitive function

Figure 2 demonstrates the impact of physical exercise on executive function and various cognitive domains. Twelve clinical and cognitive scales were used for comparison, including the Alzheimer’s Disease Assessment Scale–Cognitive Subscale (ADAS-Cog, *n* = 5) [26, 33, 34, 59, 64], Montreal Cognitive Assessment/Cognitive Abilities Screening Instrument (MoCA/CASI, *n* = 18) [7, 12, 15, 20, 27–29, 31, 32, 35–37, 40, 53–55, 63, 65], Mini-Mental State Examination (MMSE, *n* = 14) [7, 24, 26, 27, 29, 30, 36, 53, 54, 57, 58, 60, 61, 65], Trail Making Test Part A & B (TMT A & B, *n* = 11) [12, 14, 20, 28, 33, 37, 53–56, 59], Immediate Recall/Working Memory (*n* = 5) [6, 14, 25, 34, 54], Delayed Recall (*n* = 4) [12, 34, 54, 62], Forward Digit Span (*n* = 4) [12, 20, 28, 33], Backward Digit Span (*n* = 3) [20, 28, 33], Verbal Fluency (*n* = 6) [6, 20, 37, 55, 60, 62], Symbol Digit Modalities Test (SDMT, *n* = 2) [34, 65], Digit Symbol Substitution Test (DSST, *n* = 2) [14, 37], and dual-tasking (*n* = 1) [41].

Our meta-analyses indicated significant improvements in ADAS-Cog (SMD = 0.353, CI [0.081 to 0.626], *p* = 0.011), MoCA/CASI (SMD = 1.086, CI [0.700 to 1.472], *p* < 0.001), MMSE (SMD = 1.047, CI [0.658 to 1.437], *p* < 0.001), TMT A & B(SMD = 0.635, CI [0.272 to 0.999], *p* = 0.001), delayed recall (SMD = 0.318, CI [0.045 to 0.592], *p* = 0.022), forward digit span (SMD = 1.162, CI [0.006 to 2.317], *p* = 0.049), backward digit span (SMD = 1.117, CI [-0.001 to 2.235], *p* = 0.050), verbal fluency (SMD = 0.793, CI [0.366 to 1.221], *p* < 0.001), and dual-tasking (SMD = 0.588, CI [0.003 to 1.173], *p* = 0.049). In contrast, immediate recall/working memory (SMD = 0.067, CI [-0.099 to 0.259], *p* = 0.463), SDMT (SMD = 0.157, CI [-0.320 to 0.633], *p* = 0.519), and DSST (SMD = 0.071, CI [-0.129 to 0.271], *p* = 0.487) did not demonstrate significant improvement.

### Measure of methodological quality and publication bias

**Fig. 3:**
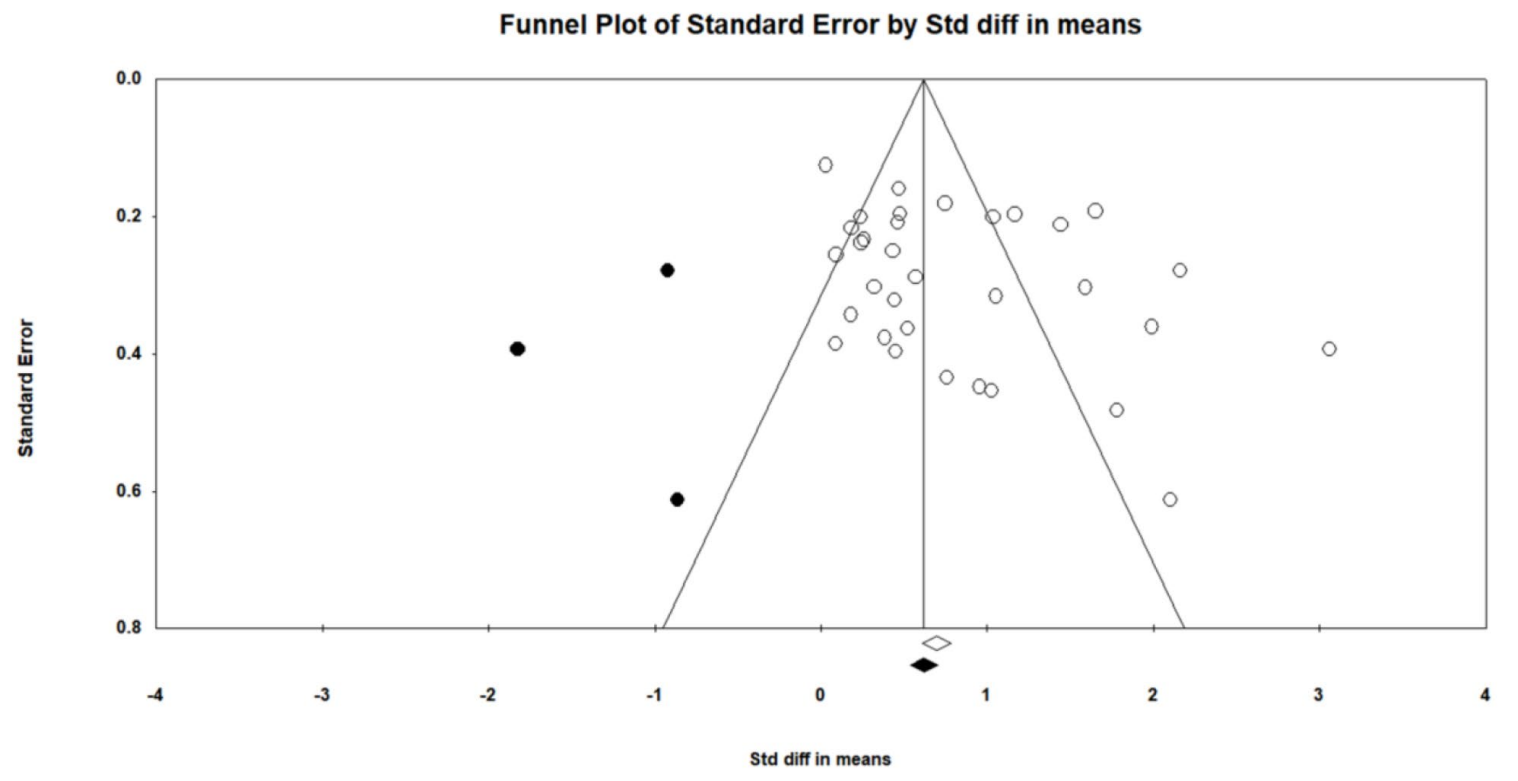
The funnel plot showing the outcomes of the Egger’s regression test for potential publication bias. White circles represent the included studies while the black circles represent the imputed “missing studies”

The methodological quality of the included studies, evaluated using the PEDro scale, was generally high, with an average score of 8 (Table 1) [66, 67]. However, most studies did not achieve blinding of both the subjects and the therapists administering the therapy. Additionally, 20 studies did not adequately report the concealment of subject group allocation, and 11 studies lacked reports on the blinding of assessors for at least one key outcome. Furthermore, Egger’s regression test indicated potential publication bias (t = 2.08, *p* = 0.045). The Duval and Tweedie’s trim and fill method revealed that the adjusted effect size, at an SMD of 0.68 [0.43 to 0.93], was slightly lower compared to the observed SMD of 0.82 [0.43 to 0.93] (as shown in Fig. 3) [68, 69].

**Table 1:** PEDro scores for each study included in this meta-analysis

Study	1	2	3	4	5	6	7	8	9	10	11	Total
Chang et al. [31]	1	1	0	1	0	0	1	0	1	1	1	**7**
Jurakic et al. [32]	1	1	0	1	0	0	0	1	1	1	1	**7**
Hsieh et al. [40]	1	0	0	1	0	0	0	1	1	1	1	**6**
Huang et al. [54]	1	1	1	1	0	0	0	1	1	1	1	**8**
Khanthong et al. [55]	1	1	0	1	0	0	1	1	1	1	1	**8**
Khattak et al. [53]	1	1	1	1	0	0	0	1	1	1	1	**8**
Law et al. [56]	1	1	0	1	0	0	1	1	1	1	1	**8**
Lee et al. [14]	1	1	1	1	0	0	1	1	1	1	1	**9**
Li et al. [20]	1	1	1	1	0	0	1	1	1	1	1	**9**
Li et al. [30]	1	0	0	1	0	0	0	1	1	1	1	**6**
Li et al. [29]	1	1	0	1	0	0	1	1	1	1	1	**8**
Lü et al. [33]	1	1	0	1	0	0	1	1	1	1	1	**8**
Liu et al. [36]	1	1	0	1	0	0	1	1	1	1	1	**8**
Mavros et al. [34]	1	1	0	1	1	0	1	1	1	1	1	**9**
Parial et al. [28]	1	1	1	1	0	0	1	1	1	1	1	**9**
Campo et al. [37]	1	1	1	1	0	0	1	1	1	1	1	**9**
Rojasavastera et al. [35]	1	1	1	1	0	0	0	1	1	1	1	**8**
Schwenk et al. [41]	1	1	1	1	0	0	1	1	1	1	1	**9**
Siu et al. [58]	1	0	0	1	0	0	0	1	1	1	1	**6**
Song et al. [15]	1	1	1	1	0	0	1	1	1	1	1	**9**
Sugano et al. [6]	1	1	0	1	0	0	0	1	1	1	1	**7**
Tao et al. [7]	1	1	0	1	0	0	1	1	1	1	1	**8**
Thaiyanto et al. [59]	1	0	0	1	0	0	1	1	1	1	1	**7**
Toots et al. [60]	1	1	1	1	0	0	1	1	1	1	1	**9**
Uysal et al. [24]	1	1	0	1	1	1	1	1	1	1	1	**10**
Varela et al. [61]	1	1	0	1	0	0	1	1	1	1	1	**8**
Vital et al. [62]	1	0	0	1	0	0	1	1	1	1	1	**7**
Xia et al. [63]	1	1	1	1	0	0	1	1	1	1	1	**9**
Yágüez et al. [25]	1	1	0	1	0	0	0	1	1	1	1	**7**
Yang et al. [26]	1	1	0	1	0	0	0	1	1	1	1	**7**
Yoon et al. [27]	1	1	0	1	0	0	0	0	1	1	1	**6**
Average												**8**

*Note* The specific 11 category descriptors are listed below

1. Eligibility criteria were specified

2. Subjects were randomly allocated to groups (in a crossover study, subjects were randomly allocated an order in which treatments were received)

3. Allocation was concealed

4. The groups were similar at baseline regarding the most important prognostic indicators

5. There was blinding of all subjects

6. There was blinding of all therapists who administered the therapy

7. There was blinding of all assessors who measured at least one key outcome

8. Measures of at least one key outcome were obtained from more than 85% of the subjects initially allocated to groups

9. All subjects for whom outcome measures were available received the treatment or control condition as allocated or, where this was not the case, data for at least one key outcome was analysed by “intention to treat”

10. The results of between-group statistical comparisons are reported for at least one key outcome

11. The study provides both point measures and measures of variability for at least one key outcome

### Exercise types

**Fig. 4:**
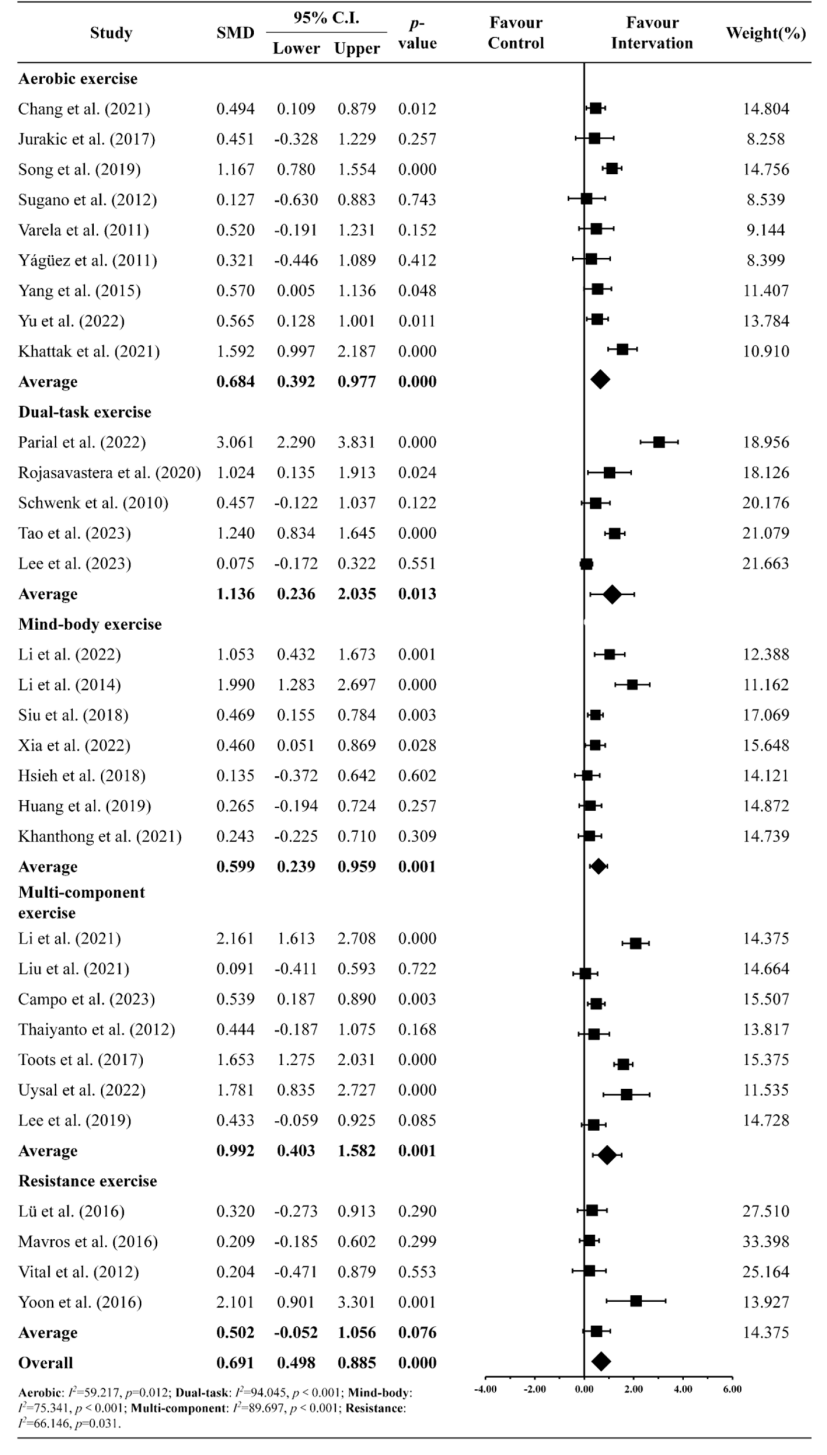
Forest plot of the exercise types on the various standardized clinical tests and assessments of cognitive function

The forest plot in Fig. 4 depicts how different types of exercise enhance executive function. Aerobic exercise emerged as the most common type (*n* = 9) [6, 15, 25, 26, 31, 32, 53, 61, 64], while resistance exercise was least common (*n* = 4) [27, 33, 34, 62]. Overall, all exercise types demonstrated a favorable impact on executive function (SMD = 0.691, CI [0.498 to 0.885], *p* < 0.001). Subgroup analyses revealed significant cognitive improvements across various exercises, including aerobic exercise (SMD = 0.684, CI [0.392 to 0.977], *p* < 0.001) [6, 15, 25, 26, 31, 32, 53, 61, 64], dual-task training (SMD = 1.136, CI [0.236 to 2.035], *p* = 0.013) [7, 14, 28, 35, 41], mind-body exercise (SMD = 0.599, CI [0.239 to 0.959], *p* = 0.001) [20, 30, 40, 54, 55, 58, 63], multi-component exercise (SMD = 0.992, CI [0.403 to 1.582], *p* = 0.001) [24, 29, 36, 37, 57, 59, 60], and resistance exercise (SMD = 0.502, CI [-0.052 to 1.056], *p* = 0.076) [27, 33, 34, 62], though no significant differences were observed between the exercise types. This was particularly evident as dual-task training exhibited the greatest enhancement in executive function, while mind-body exercise showed the least improvement.

### Exercise intensity

**Fig. 5:**
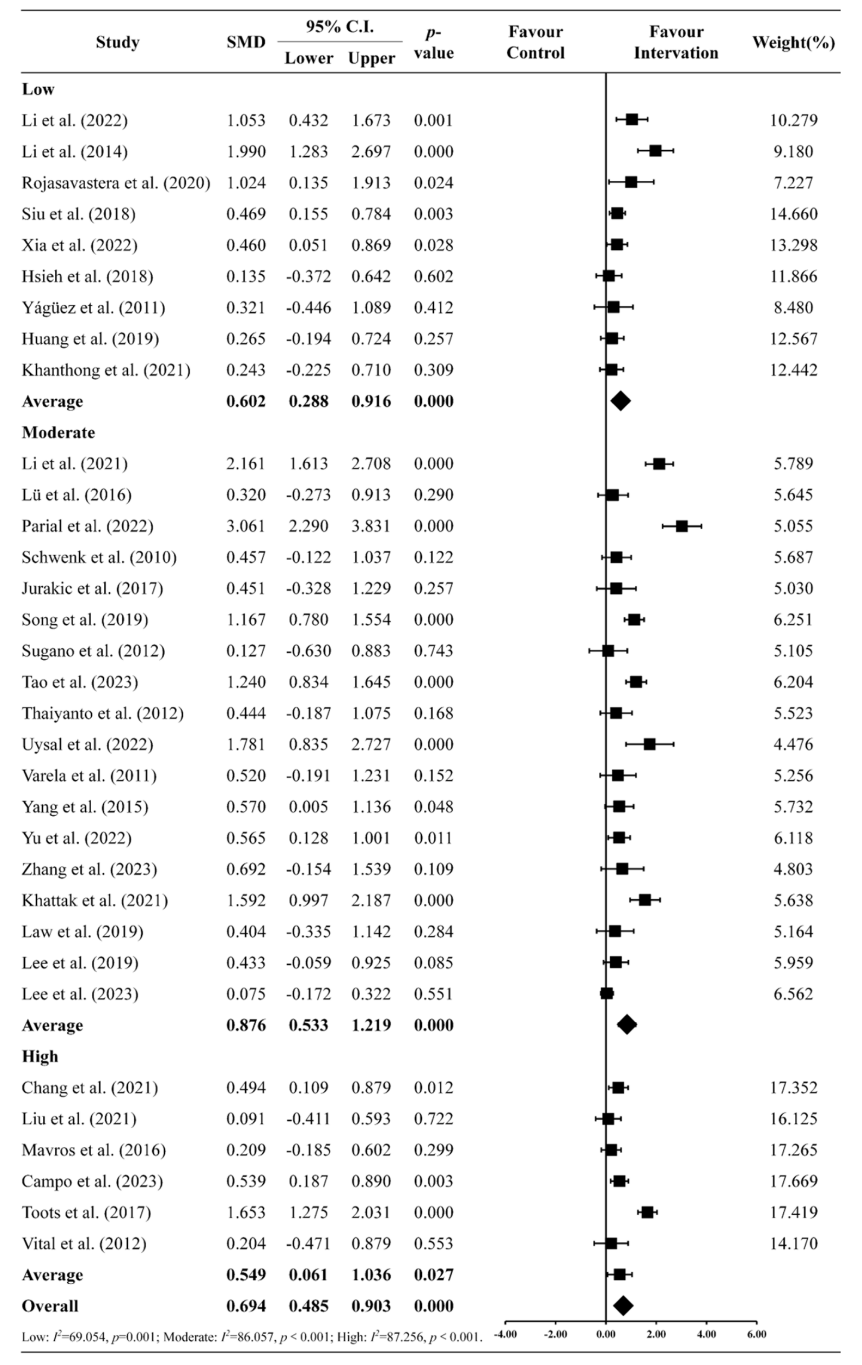
Forest plot of the exercise intensity on the various standardized clinical tests and assessments of cognitive function

Figure 5 depicts a forest plot that demonstrates the impact of exercise intensity on cognitive function, with separate groups for low- (*n* = 9) [20, 25, 30, 35, 40, 54, 55, 58, 63], moderate- (*n* = 18) [6, 7, 14, 15, 24, 26, 28, 29, 32, 33, 41, 53, 56, 57, 59, 61, 64, 65], and high-intensity (*n* = 6) [31, 34, 36, 37, 60, 62]. The study’s findings revealed significant enhancements in cognitive function across all exercise intensity levels, favoring the intervention group: low- (SMD = 0.602, CI [0.288 to 0.916], *p* < 0.001), moderate- (SMD = 0.876, CI [0.533 to 1.219], *p* < 0.001), and high-intensity (SMD = 0.549, CI [0.061 to 1.036], *p* = 0.027) exercises [7, 70], with no significant differences observed overall between the different exercise intensities.

### Exercise duration

**Fig. 6:**
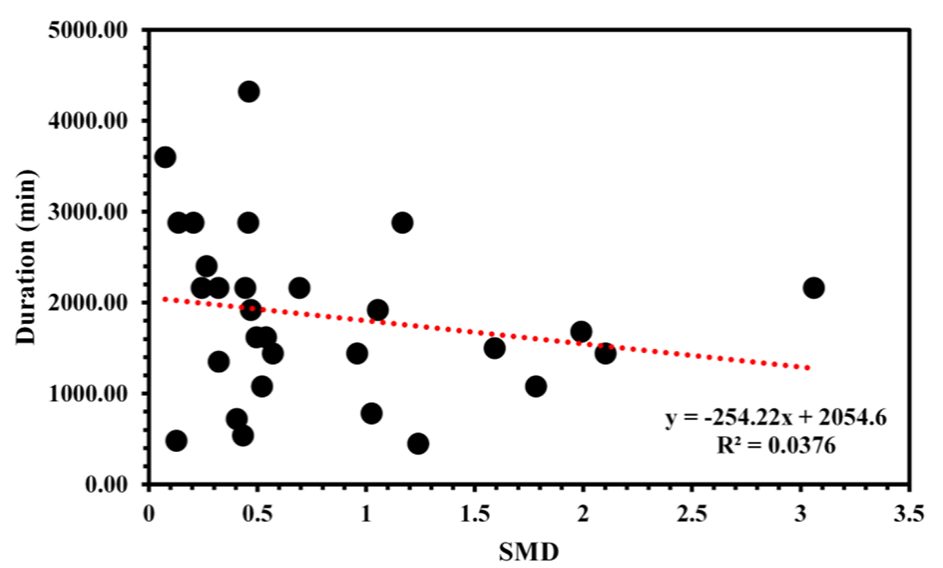
Scatter plot of exercise duration and cognitive function

Based on our meta-regression analysis, our results showed that exercise duration was not a strong predictor of SMD (R^2^ = 0.0376, *p* = 0.313) (See Fig 6).

## Discussion

In this systematic review and meta-analyses, the effects of physical exercise parameters, specifically exercise types, intensity, and duration on executive function in older adults with MCI or dementia were evaluated. The analysis produced four principal findings. First, the meta-analyses demonstrated that physical exercise positively influenced outcomes of standardized clinical tests such as the ADAS-Cog, MoCA/CASI, and MMSE, and most assessments of cognitive function, except for immediate recall, SDMT, and DSST measures. Second, all types of physical exercise (i.e., aerobic, resistance, dual-task, mind-body, and multi-component exercises) were found to enhance executive function, with dual-task exercises showing the greatest overall positive effect. Third, physical exercise performed at either low, moderate, or high-intensity resulted in significant improvements in cognitive and executive functioning, with moderate-intensity exercise yielding the greatest positive effects on executive function. Fourth, meta-regression analysis indicated no significant correlation between exercise duration and improvements in executive functioning [71]. The assessment of methodological quality and publication bias revealed that, although the overall quality of included studies was high, there was a potential for publication bias favoring studies reporting positive outcomes [72, 73]. Overall, our findings confirm the effectiveness of physical exercise in improving executive function in older adults with MCI or dementia, and highlight that careful consideration of exercise parameters, such as exercise type, intensity, and duration is essential to optimize executive function in older adults with MCI or dementia.

### Methodological quality and publication bias

A total of 35 studies met the inclusion criteria for qualitative synthesis and quantitative meta-analyses. The average overall rating of these studies on the PEDro scale was 8, indicating high methodological quality [74, 75]. However, 4 categories on the PEDro scale (Category 3 – Subject allocation was concealed, Category 5 – Blinding of all subjects, Category 6 – Blinding of therapist administering the therapy, and Category 7 – Blinding of assessors administering at least one key outcome) had more than 50% of studies fail to meet these criteria. While lack of allocation concealment and blinding of subjects, therapists, and assessors can potentially bias outcomes, it is often difficult to employ a double-blinded study design for exercise trials, unlike drug trials where placebo drugs are available. To overcome the limitations of potential bias, a large proportion of included studies used active control groups, such as light stretching and exercise [20, 55], or health education programs [15] to control for environmental and social influences on cognitive and executive function that provided an added element of experimental rigor. In addition to assessing methodological quality, our analyses indicated potential publication bias, with most studies favoring the intervention group [76]. This phenomenon of potential publication bias favoring the intervention group is common in other fields of exercise science research, reflecting a systemic issue of publishing primarily successful findings [77]. However, including studies with null or negative results is essential for a comprehensive understanding of the true effects of exercise interventions on executive function outcomes. Notwithstanding the results from the Egger’s test indicating a possibility for publication bias, the adjusted SMD still indicated that the overall effect was large and in favor of the intervention condition.

### Effects of physical exercise on measure of clinical neuropsychological and cognitive assessments

Our study demonstrated that physical exercise, regardless of type and intensity, positively impacted cognitive and executive function assessments. Specifically, the ADAS-Cog, MoCA/CASI, and MMSE demonstrated overall improvements following exercise, with the MoCA/CASI and MMSE showing the most significant enhancements compared to the ADAS-Cog. These results align with previous meta-analyses that employed similar outcome measures [78, 79]. Notably, the effects on the MoCA/CASI and MMSE were more pronounced than those on the ADAS-Cog. This is likely due to the higher number of studies utilizing MoCA and MMSE as assessment outcomes compared to ADAS-Cog. However, it is difficult to clearly ascertain why we see such large positive effects on these standardized neuropsychological assessments as they are often multi-dimensional, which assesses multiple domains of executive function to provide a composite score for diagnostic purposes.

To more accurately assess the impact of physical exercise on various cognitive functions, we conducted separate meta-analyses for each cognitive assessment. Our results revealed that physical exercise had the most significant effects on the TMT (i.e., a measure of visual searching, attention, and task-switching) and verbal fluency tests, compared to other cognitive measures such as immediate and delayed recall, forward and backward digit span, SDMT, DSST, and dual-tasking abilities [80]. While the outcomes of our findings may be influenced by the number of studies included for the TMT and verbal fluency domains, physical exercise could directly target the neural mechanisms underpinning these assessments. Both TMT and verbal fluency assessments require cognitive abilities, such as selective attention, inhibition, mental flexibility, response generation, and self-monitoring, which are likely to be specifically strengthened by physical exercise. Conversely, domains such as working memory may not be as significantly impacted. Although delayed recall, digit span, and dual-tasking also demonstrated positive effects from physical exercise, these were less pronounced due to fewer studies focusing on these areas [81, 82]. Research indicates that improvements in physical or cognitive functions are often specific to the type of training intervention used, with limited transfer to other cognitive domains [83]. Therefore, a targeted approach is crucial when selecting exercises to enhance cognitive and executive functions.

### Exercise specificity: a focus on exercise type on cognitive and executive functioning

When considering exercise type as a moderator in our meta-analyses, our results indicated that all exercise modalities significantly benefited executive function in older adults with MCI or dementia. There is now ample evidence supporting that lifestyle interventions, particularly exercise, are among the most effective means to enhance brain health and function [84]. A key consideration for exercise is how it elicits neural and/or physiological adaptations that effect positive changes in executive functioning and behavior. In the meta-analyses, we identified five commonly used exercise types: aerobic, resistance, mind-body, multi-component, and dual-tasking. Aerobic exercises accounted for the largest proportion of studies included in this review, likely due to their ease of implementation and familiarity to older participants. Aerobic exercises also confer physiological benefits that support brain and cognitive health, improving cardiovascular and cardiometabolic functioning, which leads to better control of blood pressure [85], glucose metabolism [86], and cholesterol levels [87]. This, in turn, reduces the risk of cerebral small vessel disease, a major risk factor for vascular dementia, age-related cognitive declines, and strokes [88, 89]. Studies have shown that aerobic exercise upregulates the production of neurotrophic factors, such as brain-derived neurotrophic factor (BDNF), and neurotransmitters, such as dopamine and serotonin, which are essential for supporting neuroplasticity and brain function, particularly in older adults with MCI or dementia [90, 91, 92]. In resistance and dual-tasking exercises, the mechanisms of action for improving executive functioning may differ somewhat from aerobic exercises [93]. A key objective of resistance and dual-task training is not only to enhance neuromuscular function but also to develop functional movement proficiencies. These training types are designed to simulate daily activities and incorporate cognitive skills and strategic thinking, which lead to improved cognitive functioning [94, 95]. Unlike aerobic exercise, which creates a suitable physiological environment for optimal brain functioning, resistance and dual-task training directly targets cognitive processes [7, 27, 28]. Our findings showed that dual-tasking exercises had the greatest impact on cognitive outcomes [28], followed by multi-component exercises [29].

In multi-component exercises, unlike dual-task exercises which involve simultaneous motor and/or cognitive tasks, various types of exercises are performed sequentially within a session (e.g., 20 min of aerobic followed by 20 min of resistance training) or across sessions (e.g., aerobic exercise in one session and strength training in the next), aiming to combine benefits from different exercise modalities [96, 97]. These exercises, including aerobic, resistance, and mind-body exercises, aim to improve cardiovascular and neuromuscular functions [78]. It is unsurprising that multi-component exercise yielded higher effect sizes compared to single-type exercises. From a practical standpoint, multi-component exercise programs more closely represent real-world lifestyle programs that older adults are likely to engage in. More importantly, there are likely synergistic effects from combining various exercises. For example, a combination of aerobic exercise and dual-tasking is likely to result in better cardiovascular functioning (i.e., creating an optimal physiological environment) and strengthening of neural pathways involved with executive function, which are synergistic in nature. Indeed, studies combining physical exercise and cognitive training have reported greater effects of combined physical-cognitive training, as compared to either training method performed alone [98]. Finally, mind-body exercises such as Yoga, Taichi, Qigong, and Baduanjin have also been shown to significantly affect executive function. While the criteria for what constitutes mind-body exercises is still debated, a key component of mind-body exercise focuses on meditation during movement execution, which involves coordinating between breathing, body sense and awareness, and movement execution [20, 30, 58]. It is likely that the process of learning to coordinate these actions strengthens cognitive processes such as attention regulation, inhibition, and emotional regulation that are crucial for executive function.

Overall, when considering the effects of exercise type on executive functioning in older adults, it is important to acknowledge that physical exercise as a whole is beneficial for brain health and function. However, in response to our first research question of *“Which type of physical exercise is most beneficial…”*, a key consideration is determining the needs and capacity of older adults regarding physical exercise. The current consensus indicates that most exercise interventions adopt an aerobic approach, likely due to its functional nature, ease of administration, and widespread acceptability. However, as our findings demonstrate, other types of exercise are just as beneficial, and a multi-component approach may offer greater holistic benefits to support the cognitive, psychological, and physical health of older adults with MCI or dementia [99].

### Exercise dosing: effects of exercise intensity and duration on executive functioning

Exercise intensity is another major consideration when examining the dosing effects and adaptations resulting from exercise. Like exercise type, all three exercise intensities significantly improved executive function, with moderate-intensity showing the greatest effect [70, 100]. Substantial evidence supports that even low-intensity physical activity benefits cardiovascular [78], cognitive [30], and psychological health [101]. However, the effects of physical exercise on physiological functions are often intensity-specific [102], necessitating increased intensity for greater adaptations. Although our findings revealed no significant differences in overall effect sizes among exercise intensities, studies using moderate-intensity exhibited the greatest overall effects compared to those using low or high intensities. This is consistent with previous meta-analyses where moderate exercise intensities elicited the largest effects on cognitive functioning markers [78, 103]. In acute or single-session studies, a commonly reported inverted-U relationship exists between exercise intensity and cognitive outcomes [104, 105]. These findings suggest the existence of an optimal exercise intensity range corresponding to moderate intensity [106]. This leads to optimal stimulation of psychological and physiological factors (i.e., arousal, hormone, and neurotransmitter production) that help to support cognitive functioning that, in turn, drives cognitive and behavioral changes. By contrast, low-intensity exercises are unlikely to provide sufficient stimulus to elicit physiological adaptations, while high-intensity or near-maximum exercises may induce fatigue and a heightened state of arousal (i.e., hyperfocality), which may limit the enactment and training of various cognitive processes. An additional consideration in our meta-analysis was that certain exercises, such as mind-body exercises, were mostly categorized as being low-intensity according to standard guidelines [40, 54, 55]. While this may potentially bias the distribution of exercise across the three exercise intensity categories, it also reflects the potential of such exercises, even at low intensities, to improve cognitive function in older adults with MCI or AD. Considering that older adults with MCI or AD are likely to have other comorbidities that would limit their ability to perform moderate or high intensity exercises, such low intensity exercises may offer a suitable alternative to improving cognitive functioning.

A surprising finding from our results was that we did not observe any clear relationship between exercise duration and the observed effects of the included studies. Initially, we expected a positive relationship between exercise duration and its effects, anticipating that longer exercise durations would show greater benefits. Several plausible explanations exist for this observation. First, changes in executive functioning are likely influenced by factors such as baseline executive functioning levels and age-related comorbidities (i.e., cardiovascular, metabolic, and musculoskeletal declines) in older adults [107, 108]. That is, the included studies have recruited participants of differing severity of cognitive impairments, with some studies recruiting older adults with MCI [32, 40, 41, 55], while other studies recruited AD [25, 26, 62], or a combination of both [64]. This is likely to influence the magnitude of change in executive functioning outcomes from the exercise intervention. Further, risk factors and comorbidities such as cerebral small vessel disease, diabetes, high cholesterol, and blood pressure are particularly common in older adults, which could have influenced the effects of exercise. Secondly, changes to executive functioning likely follow a time course throughout the intervention. Specifically, executive functioning improvements are typically rapid in the early stages of an intervention and plateau during later stages (i.e., diminishing returns) [54], complicating the identification of a clear association. However, this does not imply that longer interventions are counterproductive. While no clear relationships were established within our review’s constraints, longer interventions might demonstrate more pronounced effects on the long-term retention of executive functioning than immediately observed effects. Indeed, several long-term exercise RCTs have shown significant improvements and maintenance of cognitive function months after the intervention, an important outcome beyond the scope of this review [109, 110].

In response to our research questions on *“Which exercise intensity is most beneficial…”* and *“What is the relationship between exercise duration and changes in executive function…”*, our findings indicate that although all exercise intensities benefit executive functioning, moderate intensity exercises may elicit the greatest changes. However, it is important to note that the goal of physical exercise extends beyond improving executive functioning; other bodily functions may require varying levels of exercise intensity to realize potential benefits [111]. Furthermore, there appears to be no clear relationship between exercise duration and immediate changes in executive function post-intervention. However, this may be due to our inclusion criteria more than actual effects, and further investigation into the retention effects of physical exercise on executive functions is warranted.

### Gaps in the literature and future directions

Based on our results, physical exercise significantly improves executive functioning in older adults with MCI or dementia. However, several limitations within our meta-analyses should be acknowledged. First, since our review focused on the immediate effects of exercise parameters on executive function, we were unable to address the follow-up or retention of executive functioning skills resulting from physical exercise. It is likely that manipulation of exercise parameters may also influence the retention of executive functioning, which is a significant aspect of neurorehabilitation. Second, we were unable to distinguish our findings based on whether physical exercises were performed individually or in a group setting. Group settings may be influenced by social factors, such as interpersonal interactions, which could affect executive function outcomes. Finally, although we analyzed the relationship between exercise duration and observed effects, other factors such as exercise volume (the total amount of work done across all sessions) may also influence executive function outcomes. However, standardizing the calculation of exercise volume across studies is challenging, given the diversity in the range, type, and nature of exercises performed.

The findings from this systematic review and meta-analysis provide conclusive evidence that physical exercise benefits executive function in older adults with MCI or dementia. While the cognitive benefits of physical exercise are clear, a deeper understanding of the neurophysiological mechanisms underlying these effects is necessary. Particularly, understanding the synergistic effects of combining various exercise modalities to augment executive function is a critical gap that needs addressing. Additionally, the success of any exercise intervention critically depends on adherence levels to exercise programs in real-world settings. Understanding the social and cultural beliefs towards physical exercise is crucial to identify barriers and facilitators of exercise adherence. Given that social barriers and facilitators are often influenced by sociodemographic factors, successful exercise program implementation must consider the socio-cultural context and beliefs where these programs are conducted.

## Conclusion

Our systematic review and meta-analyses align with existing literature regarding the impact of physical exercise on executive function in older adults with MCI and dementia. It was noted that all forms of physical exercise are advantageous, emphasizing the importance of customizing exercise regimens to suit the individual needs and abilities of older adults. Understanding the mechanisms through which exercise enhances executive function is crucial for tailoring exercise programs for optimal results. While varying intensities of physical exercise have positive effects on cognitive function, moderate intensity levels may be most effective in improving executive functioning. Future research should delve deeper into the neurophysiological mechanisms and combined effects of different exercise modalities on executive function in older adults with MCI or dementia.

### Electronic supplementary material

Below is the link to the electronic supplementary material.

Supplementary Material 1

## Data Availability

All data and materials pertaining to this systematic review and meta-analysis is available upon request via the corresponding author.
