# Supplementary material for: Identifying exercise and cognitive intervention parameters to optimize executive function in older adults with mild cognitive impairment and dementia: a systematic review and meta-analyses of randomized controlled trials
Source: Eur Rev Aging Phys Act. 2024 Aug 30;21:22. doi: 10.1186/s11556-024-00357-4 (PMC11363393; doi:10.1186/s11556-024-00357-4)
Supplement: Supplementary file 1 — Supplementary Material 1 [file 11556_2024_357_MOESM1_ESM.docx]

**Table S1** Studies included in systematic review and meta-analyses

| **Study author** | **Country and Year of publication** | **Participant (number and condition)** | **Control** | **Intervention (Exercise Parameters) (exercise intensity)** | **Executive Function measure** | **Outcomes** |
| --- | --- | --- | --- | --- | --- | --- |
| Chang et al. | China  2021 | 225 older adults with cognitive decline, aged ≥ 60 years | Standard/usual care | Chinese square dance; three times a week for 30 min; high intensity | 1. MoCA;  2. MCS. | 1. MoCA scores showed a significant improvement in the intervention group compared to the control group;  2. MCS scores of the intervention group improved compared to the control group. |
| Jurakic et al. | Switzerland 2017 | 28 older adults with MCI, aged 66-78 years | Pilate’s training (three times a week through 8 weeks) | HUBER training; three times a week through 8 weeks; moderate intensity | MoCA | Both groups showed significant improvements in the overall MoCA score. |
| Hsieh et al. | Taiwan  China  2018 | 60 older adults with MCI, aged 78.2 ± 7.7 years, ranged 60-96 years | Usual daily physical activities during the 6-month period | VR-based Tai Chi exercise program; 60 min group session twice weekly for 6 months; low-intensity | CASI | Average movement accuracy score of 3 months significantly predicted improvement in the total CASI score. However, there was no statistical significance at 6 months. |
| Huang et al. | China  2019 | 80 older people with mild dementia, aged ≥ 60 years | Routine treatments and personalized daily care | Tai Chi program; three times a week for 10 months; low intensity | 1. MMSE;  2. MoCA;  3. TMT;  4. WHO-UCLA-AVLT (delayed recall);  5. WHO-UCLA-AVLT (Immediate recall). | 1. There was no such improvement in the MMSE;  2. There was an increasing trend in the intervention group in the MoCA test over the 10-month study period;  3. TMT score of Tai Chi group decreased, but an increase in CG group;  4. There was no statistically significant time or group difference in the WHO-UCLA-AVLT (delayed recall and Immediate recall). |
| Khanthong et al. | Thailand  2021 | 71 older adults with MCI, aged 50-80 years | No exercise | Ruesi Dadton exercise; 60 min, 3 times/week for 12 weeks; low intensity | 1. MoCA;  2. VF;  3. TMT-A;  4. TMT-B. | 1. MoCA scores were significantly improved in both the RSD and control groups;  2. RSD group showed significant improvements for VF test;  3. RSD group showed significant improvements for TMT-B, but there was no significant difference in TMT-A. |
| Khattak et al. | Pakistan  2021 | 60 older adults, aged 62.49 ± 1.82 years | Gentle active range of motion exercises and stretching for 5-7 cycles for 5 days/week for 6 weeks | Aerobic exercise program; 40-50 min/day, 5 days a week for 6 weeks; moderate intensity | 1. MMSE;  2. MoCA;  3. TMT-A;  4. TMT-B. | Experimental group showed improvement in MMSE, MoCA, TMT-A and B (p < 0.05) after 6 weeks of intervention with aerobic exercise. |
| Law et al. | China  2019 | 59 older adults with MCI, aged ≥ 60 years | Normal activity or exercise pattern during the 8-weeks | Functional task exercise/Cognitive training/Exercise training groups: 12 sessions for 8 weeks; 40-60 min; moderate intensity | 1. NCSE;  2. CVVLT;  3. TMT-A;  4. TMT-B. | 1. The functional task exercise group demonstrated significant within-group improvements in all outcomes; Both the cognitive training group and the exercise training group did not show any significant within-group differences. The control group showed a significant decrease in the NCSE composite score;  2. The functional task exercise group had significant between-group differences in the CVVLT total free recall score;  3. The results showed an approaching significant difference in the TMT-A and TMT-B scores. |
| Lee et al. | Korea  2019 | MCI: 33 older adults with MCI, aged 73.88 ± 7.46 years;  CNE: 32 older adults, aged 76.5 ± 5.6 years | 60–80 min per session for eight weeks once weekly | 60–80 min per session for eight weeks once weekly; moderate intensity | MMSE | 1. The MMSE of MCI group showed statistically significant improvement;  2. The MMSE of CNE group showed statistically significant improvement in the cognitive and motor function evaluation except MMSE. |
| Lee et al. | Japan  2023 | 280 older adults with MCI, aged ≥ 70 years | Three educational courses within 10 months | A weekly exercise course with a total of 40 sessions within 10 months; moderate intensity,  Mentally stimulating social activities twice per month; 14 times in total | 1. TMT-A;  2. TMT-B;  3. DST;  4. Logical memory immediately;  5. Logical memory recall;  6. Logical memory recognition;  7. Word list memory tasks immediately;  8. Word list memory tasks recall;  9. Word list memory tasks recognition. | 1. TMT-A scores increased in both intervention and control groups;  2. There were no differences in the TMT-B between the groups;  3. DST test scores significantly increased in intervention groups, but not in the control group;  4. Logical memory immediately increased in both intervention and control groups;  5. Recall logical memory scores increased in both intervention and control groups;  6. Recognition logical memory scores significantly increased in intervention groups at 10 months, but not in the control group;  7. Word list memory tasks immediately increased in both intervention and control groups;  8. Word list memory tasks recall increased in intervention groups, but not in the control group;  9. Word list memory tasks recognition had no differences in both intervention and control groups. |
| Li et al. | USA  2022 | 69 older adults with MCI, aged 74.6 years | Stretching group: 60-minute online exercise session via Zoom, twice weekly for 16 weeks | Cognitively enhanced/Standard Tai Ji Quan: 60 min online exercise session via Zoom, twice weekly for 16 weeks; low intensity | 1. MoCA;  2. TMT-B;  3. DST-F;  4. DST-B;  5. VF. | 1. MoCA scores of the cognitively enhanced and standard Tai Ji Quan increased, but no evident change in the stretching group;  2. TMT-B of the cognitively enhanced and standard Tai Ji Quan decreased, but no evident change in the stretching group;  3. DST-F of the cognitively enhanced and standard Tai Ji Quan increased, but no evident change in the stretching group;  4. DST-B of the cognitively enhanced and standard Tai Ji Quan increased, but the stretching group decreased;  5. VF of the cognitively enhanced and standard Tai Ji Quan increased, but no evident change in the stretching group. |
| Li et al. | USA  2014 | 46 older adults with MCI, aged ≥ 65 years | Usual daily physical activities | Tai Ji Quan; 60-min group session twice weekly for 14 weeks; low intensity | MMSE | Tai Ji Quan group exhibited significant improvements in MMSE scores, but no within-group pre-to-posttest change in the control group. |
| Li et al. | China  2021 | 90 older adults with MCI, aged ≥ 65 years | Regular  community health instruction once a month for 6 months | Multi-component exercise training once a month for 6 months; moderate intensity | 1. MMSE;  2. MoCA. | 1. MMSE scores of the intervention group increased over time, while the score of the control group gradually decreased over time;  2. MoCA scores of the intervention group increased significantly over time, while the score of the control group gradually decreased over time. |
| Liu et al. | China  2020 | 80 older adults with dementia, aged ≥ 65 years | Four aerobic training per week for 4 weeks | Strength training; 4 times per week for 4 weeks; high intensity | 1. MMSE;  2. MoCA. | 1. Both the strength training group and the aerobic training group showed an increase in MMSE scores after 4 weeks;  2. Both the strength training group and the aerobic training group showed an increase in MoCA scores after 4 weeks. |
| Lü et al. | China  2016 | 45 older adults with MCI, aged ≥ 65 years | Regular lifestyle routine without starting any new exercise activities | Dual-task gait training: 3 times per week over 12 weeks in a 60-minute momentum-based dumbbell-training class; moderate intensity | 1. ADAS-Cog;  3. DST-F;  4. DST-B;  4. TMT-B. | 1. DTG showed significant improvement in the ADAS-Cog compared to those in the CG;  2. Both the DTG and the CG group had a significant within-group improvement in DST-F;  3. Both the DTG and the CG group had a significant within-group improvement in DST-B;  4. Both the DTG and the CG group showed an increase in TMT-B. |
| Mavros et al. | Australia  2016 | 100 older adults with MCI, aged ≥ 55 years | Sham-exercise Training | Progressive Resistance Training; 2 to 3 days per week for 6 months; high intensity | 1. ADAS-Cog;  2. Global domain;  3. WAIS-III Similarities;  4. WAIS-III Matrices;  5. Category fluency;  6. COWAT;  7. Executive domain;  8. List Learning Memory Sum;  9. BVRT;  10. Immediate Memory I;  11. Delayed Memory II;  12. Memory domain;  13. Speed and attention: Symbol Digit Modalities Test. | 1. PRT and Sham-Ex groups significantly improved ADAS-Cog;  2. Global domain scores of PRT and Sham-Ex groups increased;  3. WAIS-III Similarities scores of PRT and Sham-Ex groups increased;  4. WAIS-III Matrices scores of PRT increased, while the score of the Sham-Ex group decreased;  5. Category fluency scores of PRT increased, while the score of the Sham-Ex group decreased;  6. COWAT scores of PRT and Sham-Ex groups increased;  7. Executive domain scores of PRT and Sham-Ex groups increased;  8. List Learning Memory Sum scores of PRT and Sham-Ex groups increased;  9. BVRT scores of PRT increased, while the score of the Sham-Ex group decreased;  10. Immediate Memory I scores of PRT and Sham-Ex groups decreased;  11. Delayed Memory II scores of PRT decreased, while the score of the Sham-Ex group increased;  12. Memory domain scores of PRT and Sham-Ex groups decreased;  13. Symbol Digit Modalities Test of PRT and Sham-Ex groups increased. |
| Parial et al. | China  2022 | 51 older adults with MCI, aged ≥ 55 years | Leisure activities for 12 weeks (Thrice-weekly for 60 min) | Dual-task Zumba Gold for 12 weeks; Thrice-weekly on nonconsecutive days for 60 min; moderate intensity | 1. MoCA;  2. TMT-B;  3. DST-F;  4. DST-B;  5. MoCA-MIS. | 1. MoCA score of the IG group showed a more significant increase compared to the CG group;  2. TMT-B score of the IG group showed a more significant decrease compared to the CG group;  3. DST-F scores increased in both IG and CG groups;  4. DST-B score of IG increased, but the CG group decreased;  5. MoCA-MIS score of IG increased, but the CG group decreased; |
| Campo et al. | Colombia  2023 | 132 older adults with MCI, aged ≥ 65 years | Manual activities for 12 weeks with 3 sessions per week, with a duration of 45 min per session | High-intensity functional training; 12 weeks with 3 sessions per week, 45 min per session; high intensity | 1. MoCA;  2. D2;  3. TMT-A;  4. TMT-B;  5. DSST;  6. VF. | 1. Both the IG and the CG group had a significant within-group improvement in MoCA scores;  2. D2 scores of the IG and the CG group increased significantly;  3. Both TMT-A and TMT-B scores of the IG and the CG group decreased over time;  4. DSST scores of the IG and the CG group decreased over time;  5. VF scores of the IG group showed a more significant decrease compared to the CG group. |
| Rojasavastera et al. | Thailand  2020 | 33 older adults with MCI, aged 60-80 years | No training program | Action observation with gait training OR Gait training alone0; 12 sessions with 2 or 3 sessions weekly for 2 months; low intensity | MoCA | MoCA scores of the AOGT, GT and CT groups all increased over time, with the AOGT group showing the highest increase. |
| Schwenk et al. | Germany  2010 | 61 older adults with dementia, aged ≥ 65 years | 2 times a week for 1 h of supervised motor placebo group training | Specific dual-task training OR additional progressive resistance-balance OR functional-balance training; 2hr twice per week for 12 weeks; moderate intensity | 1. Serial 2 forward;  2. Serial 3 backward;  3. Motor (gait speed) cognitive performance with serial 2 forward;  4. Motor (gait speed) cognitive performance with serial 3 backward. | 1. Serial 2 forward score of the IG group showed a more significant increase compared to the CG group;  2. Serial 3 backward score of the IG group showed a more significant increase compared to the CG group;  3. Motor (gait speed) cognitive performance with serial 2 forward score of the IG group showed a more significant increase compared to the CG group;  4. Motor (gait speed) cognitive performance with serial 3 backward score of the IG group showed a more significant increase compared to the CG group. |
| Siu et al. | China  2018 | 160 older adults with MCI, aged ≥ 60 years | Usual care group and no exercise training | Tai Chi program; 16-week, 2 sessions/week, each session was 1hr; low intensity | MMSE | IG showed greater improvement in CMMSE scores than the CG. |
| Song et al. | China  2019 | 120 older adults with MCI, aged ≥ 60 years | 16-week health education program (eight 45-minute sessions per week) | Aerobic exercise; 16-week exercise with three 60-minute group training sessions per week; moderate intensity | MoCA | IG had significantly greater improvement in their MoCA score compared with CG. |
| Sugano et al. | Japan  2012 | 14 older adults with MCI, aged 73.9 ± 5.8 years | Activity sessions | Aerobic training program; once per week, 1hr per session for 2 months; moderate intensity | 1. Abstract reasoning;  2. Attention;  3. Memory;  4. VF;  5. Visuospatial function. | 1. Abstract reasoning scores of the IG and the CG groups increased;  2. Attention scores of the IG and the CG groups increased;  3. Memory scores of the IG and the CG groups increased;  4. VF score of the IG group increased, but the CG group decreased;  5. Visuospatial function score of the IG group increased, but no difference in the CG group. |
| Tao et al. | China  2023 | 103 older adults with MCI, aged 74.45 ± 8.61 years | Health education programs | Physical movement training and integrated cognitive training; once every fortnight for 12 weeks, 60–90 min per session; moderate intensity | 1. MMSE;  2. MoCA. | 1. MMSE scores of IG group increased significantly over time compared to CG group;  2. Significant upward trend in the total MoCA score over time in IG compared with CG, with a significant difference in the MoCA score between the two groups. |
| Thaiyanto et al. | Thailand 2021 | 40 older women with MCI, aged ≥ 60 years | Routine lifestyle | Multi-component exercise program; 3 times a week for 12 weeks, 60 min per session; moderate intensity | 1. ADAS-Cog;  2. TMT-A;  3. TMT-B. | 1. There were no significant differences in ADAS-Cog;  2. IG demonstrated significantly better performance of TMT part A than the CG;  3. TMT-B score of IG increased over time, but the score of CG decreased. |
| Toots et al. | Sweden  2017 | 186 older adults with MMSE score of 15, aged ≥ 65 years | Structured activities | Functional exercise program; 40 sessions for 4 months; high intensity | 1. MMSE;  2. VF. | 1. Exercise reduced MMSE scores over time, and the IG group has a better reduction effect;  2. Exercise reduced MMSE scores over time, and the IG group has a better improving effect. |
| Uysal et al. | Turkey  2022 | 48 older adults with MCI, age ≥ 60 years | Solely lower extremity strengthening exercises (CG): three days a week for 12 weeks, 36 sessions | Aerobic plus lower extremity strengthening exercises OR  Dual-task training plus lower extremity strengthening exercises OR Aerobic exercise, dual-task training and lower extremity strengthening exercises; 36 sessions for 12 weeks; moderate intensity | MMSE | The MMSE of the AG, DG, and ADG groups improved significantly (p < 0.05), with a strong effect size (d = 0.83). The greatest change in MMSE scores was noticed in the ADG group (p < 0.001). |
| Varela et al. | Spain  2011 | 48 older adults with MCI, age ≥ 65 years (78.3 ± 9.5 years) | Recreational activities | Group A (aerobic exercise at 40% of heart rate reserve) OR Group B (aerobic exercise at 60% of heart rate); three 30 min sessions a week for three months; moderate intensity | MMSE | MMSE scores of groups A and B increased slightly, but MMSE score of group C decreased. |
| Vital et al. | Brazil  2012 | 34 older adults with mild-to-moderate stage of AD (Social gathering group: 77.6 ± 6.5 years; Weight training group: 78.2 ± 7.3 years) | Social gathering group: activities such as group dynamics, relaxation, short walks, reading, poetry, musical activities, painting, movies, mimes and recreational activities | Weight training group;  12 weeks exercise training, three times a week, 1hr per session; high intensity | 1. Identification;  2. Incidental memory;  3. Immediate memory;  4. Learning;  5. Delayed recall;  6. Recognition;  7. CDT;  8. VF. | 1. There were no significant differences in identification scores between TG and SGG;  2. Incidental memory score of SGG group demonstrated within-group improvements, but no obvious differences within-group in TG;  3. Immediate memory score of SGG group showed within-group reducing trend, but TG group showed within-group increasing trend;  4. Learning scores of SGG and TG groups showed the same increasing trend;  5. There were no significant difference in delayed recall of SGG group, but delayed recall score of TG group reduced;  6. There were no significant difference in recognition of SGG group, but delayed recall score of TG group increased;  7. There were no significant differences in CDT between TG and SGG, both TG and SGG showed slight within-group increasing trend;  8. VF score of SGG group demonstrated within-group decreasing trend, but TG score showed increasing trend. |
| Xia et al. | China  2022 | 135 older adults with MCI, aged ≥ 60 years | Original lifestyle and health education programs (one session per 8 weeks) | Baduanjin; 24 weeks 60 min sessions, 3 days per week; low intensity  24 weeks of brisk walking at a corresponding community centre; 60 min per session, 3 sessions per week; low intensity | MoCA | MoCA score of the BDJ group was significantly higher than that of the UPA group (P < 0.05). |
| Yágüez et al. | UK  2011 | 27 older adults with AD, including exercise group (70.5 ± 8 years) and control group (70.5 ± 8 years) | Standard care group | Brain gym; 6 weeks, with weekly sessions of 2 h and a 30 min break; low intensity | 1. Motor control;  2. Matching to sample simultaneous;  3. Matching to sample delayed;  4. Paired associate learning: total errors;  5. Working memory number of errors;  6. Pattern recognition total correct;  7. Rapid visual information processing: hit rate. | 1. There were no significant difference in motor control between the two groups;  2. Matching to sample simultaneous of IG showed within-group increasing trend, but TG showed a significant reducing trend;  3. Matching to sample delayed of IG and CG showed slight within-group increasing trend;  4. Both IG and CG groups showed an increase in paired associate learning;  5. Both IG and CG groups showed an decrease in paired associate learning;  6. Pattern Recognition Memory tests two groups did not differ significantly at baseline, but IG was significantly better in the pattern recognition test after training;  7. Both IG and CG groups showed same trend towards improvement in the hit rate. |
| Yang et al. | China  2015 | 50 older adults with CI, aged 50-80 years | Health education for 3 months | Aerobic exercise cycling training; 40 min/day, 3 days/week for 3 months; moderate intensity | 1. MMSE;  2. ADAS-Cog. | 1. Aerobic group showed an increasing trend in MMSE score, while MMSE scores decreased significantly after 3 months;  2. ADAS-Cog score was significantly decreased (P<0.05) in aerobic group, but no significant difference in control group. |
| Yoon et al. | Korea  2016 | 70 older adults with MCI, aged > 65 years | Balance and toning exercises | High-speed power training (high intensity)/Low-speed strength training (low intensity); 1hr exercise program, twice a week for 12 weeks | 1. MMSE;  2. MoCA. | HSPT and LSST showed similar clinically significant improvements in MMSE and MoCA, whereas the CON group showed a significant decrease in MMSE and MoCA scores. |
| Yu et al. | China  2022 | 34 older adults with MCI, aged ≥ 50 years | Usual daily activities during the study period | Tai Chi; 3 sessions of 60-min for 24 weeks (low intensity);  Conventional fitness training; 3 sessions of 60-min fitness training per week for 24 weeks; moderate intensity | 1. MoCA;  2. Delay Recall Test;  3. DST;  4. TMT-A;  5. TMT-B. | 1. TC and EX groups showed significant improvements in MoCA scores compared with CON at both mid- and post-assessments (both P < 0.001);  2. Both interventions were associated with significant improvements in the delay recall test compared with CON;  3. Both EX and TC demonstrated profound improvements in the length of the DST-F compared to CON at post-assessment, but no significant differences in the length and score of the DST-B;  4. No significant differences in TMT-A scores;  5. At mid-assessment, there were no significant differences in TMT-B difference scores. |
| Yu et al. | USA  2021 | 96 older adults with AD, aged ≥ 66 years (77.4 ± 6.8 years) | Stretching for 20–50 minutes, 3 times a week for 6 months | Cycling; 20–50 min a session, three times a week for 6 months; moderate intensity | 1. ADAS-Cog;  2. Memory;  3. Executive function;  4. Attention;  5. Processing speed;  6. Language;  7. Global cognition. | There were no differences in the 6-month rate of change in ADAS-Cog, memory, executive function, attention, processing speed, language, or global cognition. |
| Zhang et al. | China  2023 | 42 older adults with MCI, aged 60-80 years | Monthly health science knowledge dissemination activities | Traditional  Chinese exercise combined with rhythm training group; 60mins per session, 3 sessions a week for 12 weeks; moderate intensity  Walking group; 60-mins per session, 3 sessions a week for 12 weeks; moderate intensity | 1. MMSE;  2. MoCA;  3. SDMT. | 1. There was small change between the 3 groups after 6 weeks of intervention, and no significant difference between the groups, but there was a significant difference in the MMSE test between the TCE + RTG and CG after 12 weeks (P < .05);  2. The MoCA score of the TCE + RTG improved over time, but slight difference in MoCA within TCE + RTG and CG;  3. There was a small improvement in the SDMT of the TCE + RTG and CG, while SDMT of the CG decreased. |

IG- Intervention group; CG- Control group; MoCA- Montreal cognitive assessment; MMSE- Mini-mental state examination; MCS- Mental component summary; CASI- Cognitive abilities screening instrument; VF- Verbal fluency; TMT- Trail making test part; NCSE- Neurobehavioral cognitive status examination; CVVLT- Chinese version verbal learning test; MMSE-DS- Mini-mental state examination for dementia screening; ADAS-Cog- Alzheimer’s Disease Assessment Scale–Cognitive Subscale; BVRT- Benton visual retention test; DST-F- Digit symbol substitution-forward; DST-B- Digit symbol substitution-backward; DSST- Digit symbol substitution test; CDT- Clock drawing test; SDMT- Symbol digit modalities test; COWAT- Controlled oral word association test ; WAIS- Wechsler adult intelligence scale; D2- Selective attention and concentration.
